# Radiation‐Induced Tracheal Chondroradionecrosis: Bronchoscopic Diagnosis and Intralesional Triamcinolone Management

**DOI:** 10.1002/rcr2.70578

**Published:** 2026-04-07

**Authors:** Venkatkiran Kanchustambham

**Affiliations:** ^1^ Department of Pulmonary and Critical Care Medicine, Sanford Health; Department of Internal Medicine University of North Dakota School of Medicine and Health Sciences Fargo North Dakota USA

**Keywords:** bronchoscopy, chondroradionecrosis, hyperbaric oxygen, radiation injury, trachea

## Abstract

Radiation‐induced tracheal chondroradionecrosis is a rare, life‐threatening complication presenting with circumferential exposed cartilage. Bronchoscopic identification, avoidance of thermal modalities, intralesional triamcinolone injection at viable–necrotic mucosal margins, and hyperbaric oxygen referral represent a practical management strategy when surgical intervention is unavailable.

## Graphical abstract

Radiation‐induced tracheal chondroradionecrosis is a rare and potentially fatal complication of thoracic irradiation. Bronchoscopy reveals circumferential exposed cartilage with mucosal sloughing. A bronchoscopic management approach using intralesional triamcinolone injection and hyperbaric oxygen referral is demonstrated.

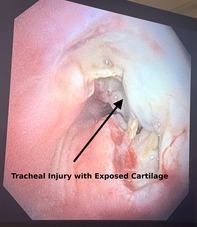

A patient with prior thoracic radiation for lung cancer presented with progressive dyspnea and cough as the predominant symptoms. One instance of possible exertional stridor was noted in primary care documentation; however, stridor was not reproducible or observed during evaluation in either the pulmonology or oncology clinic.

The patient's oncologic and treatment history is as follows. He underwent craniotomy and SBRT (2700 cGy in three fractions) to a solitary brain metastasis, completed April 9, 2021, followed by chemo‐immunotherapy, which was discontinued in 2022 due to intolerance. Subsequent PET imaging demonstrated disease progression in the right upper lobe and mediastinum, prompting consolidative thoracic radiotherapy (6000 cGy in 15 fractions to the right lung and mediastinum), completed July 16, 2024. A CT chest obtained in September 2025—approximately 14 months after radiotherapy completion—demonstrated a normal tracheal lumen with intact mucosal lining and cartilaginous architecture, establishing the pre‐injury radiographic baseline (Figure 1A). A CT chest performed on February 16, 2026 (~19 months post‐radiotherapy) first demonstrated tracheal wall changes consistent with chondroradionecrosis, with an exposed cartilage ring (Figure 1B) and circumferential tracheal injury (Figure 1C,D). This latency of approximately 19 months is consistent with the characteristically delayed presentation of radiation‐induced chondroradionecrosis [1].

**FIGURE 1:**
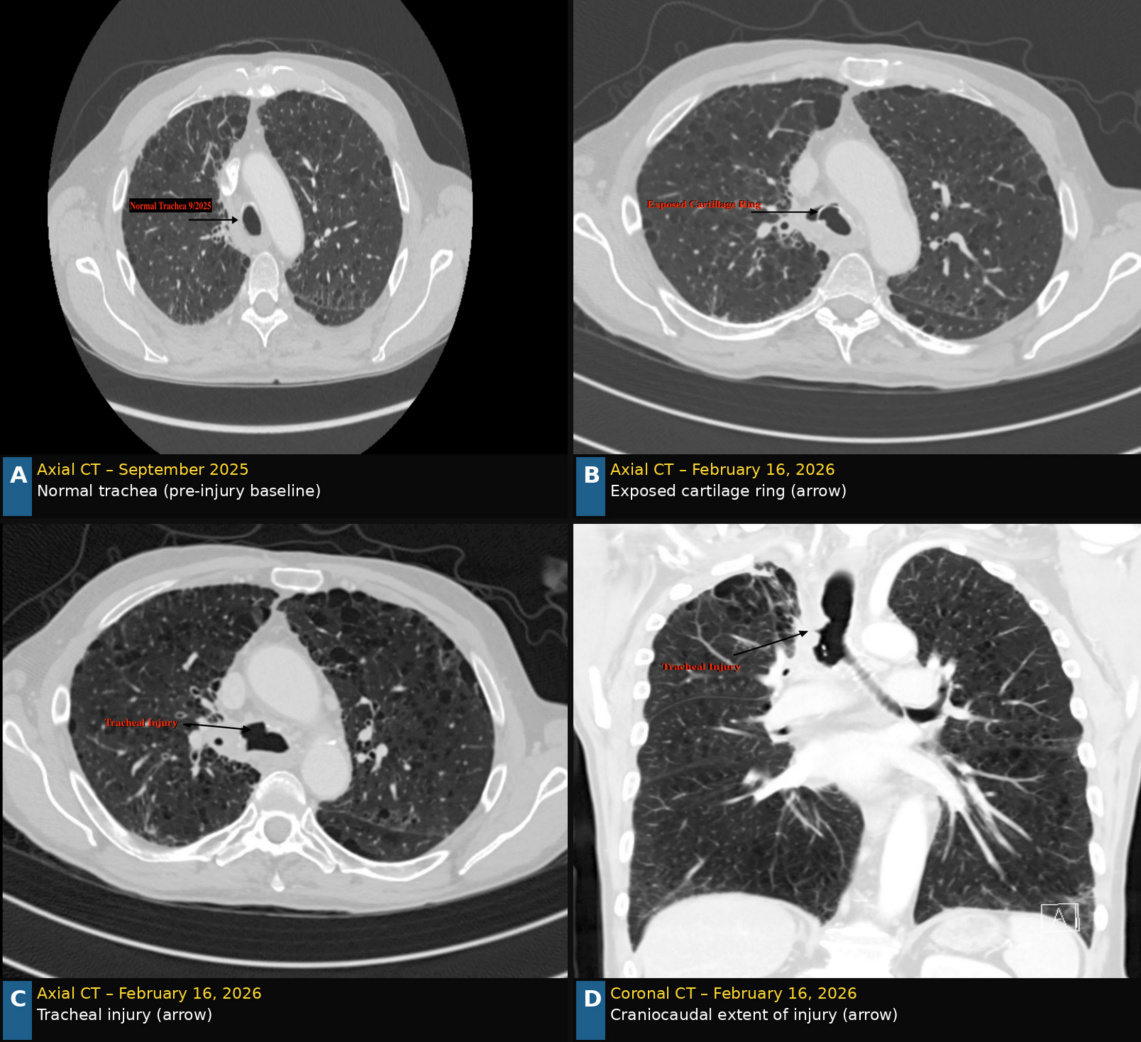
CT chest imaging demonstrating temporal evolution of radiation‐induced tracheal chondroradionecrosis. (A) Axial CT chest, September 2025: Normal tracheal lumen with intact mucosal lining and cartilaginous architecture, approximately 14 months after completion of consolidative thoracic radiotherapy (6000 cGy in 15 fractions, completed July 16, 2024), serving as the pre‐injury radiographic baseline. (B) Axial CT chest, February 16, 2026 (~19 months post‐radiotherapy): Exposed tracheal cartilage ring (arrow), representing the first CT evidence of chondroradionecrosis. (C) Axial CT chest, February 16, 2026: Circumferential tracheal injury with mucosal disruption and paratracheal soft tissue changes (arrow). (D) Coronal CT reconstruction, February 16, 2026: Craniocaudal extent of tracheal wall involvement (arrow). Plain chest radiographs obtained at presentation were non‐diagnostic; MRI of the airway was not performed.

Flexible bronchoscopy, performed on March 4, 2026, demonstrated circumferential mucosal sloughing with exposed, devitalized tracheal cartilage rings involving the right anterior, right lateral, and left lateral walls—consistent with radiation‐induced chondroradionecrosis (Figure 2). The posterior membranous wall was partially spared. Purulent secretions were gently aspirated and sent for culture. Intralesional triamcinolone acetonide (40 mg/mL diluted 1:1 with normal saline, 20 mg/mL working solution; 0.3–0.4 mL per injection site across 4–5 circumferential sites; total dose 40 mg) was administered at viable–necrotic mucosal junctions using a sclerotherapy needle. Thermal modalities were strictly avoided. The bronchoscopic findings and injection technique are demonstrated in Video 1. Hyperbaric oxygen and systemic PENTOCLO protocol (pentoxifylline + vitamin E) were initiated post‐procedure [2]. Given the extent of circumferential tracheal injury and the potential need for airway scaffolding, the patient was referred to the interventional pulmonology department at Mayo Clinic Rochester for evaluation for silicone stent placement via rigid bronchoscopy, a procedure not available at our institution.

**FIGURE 2:**
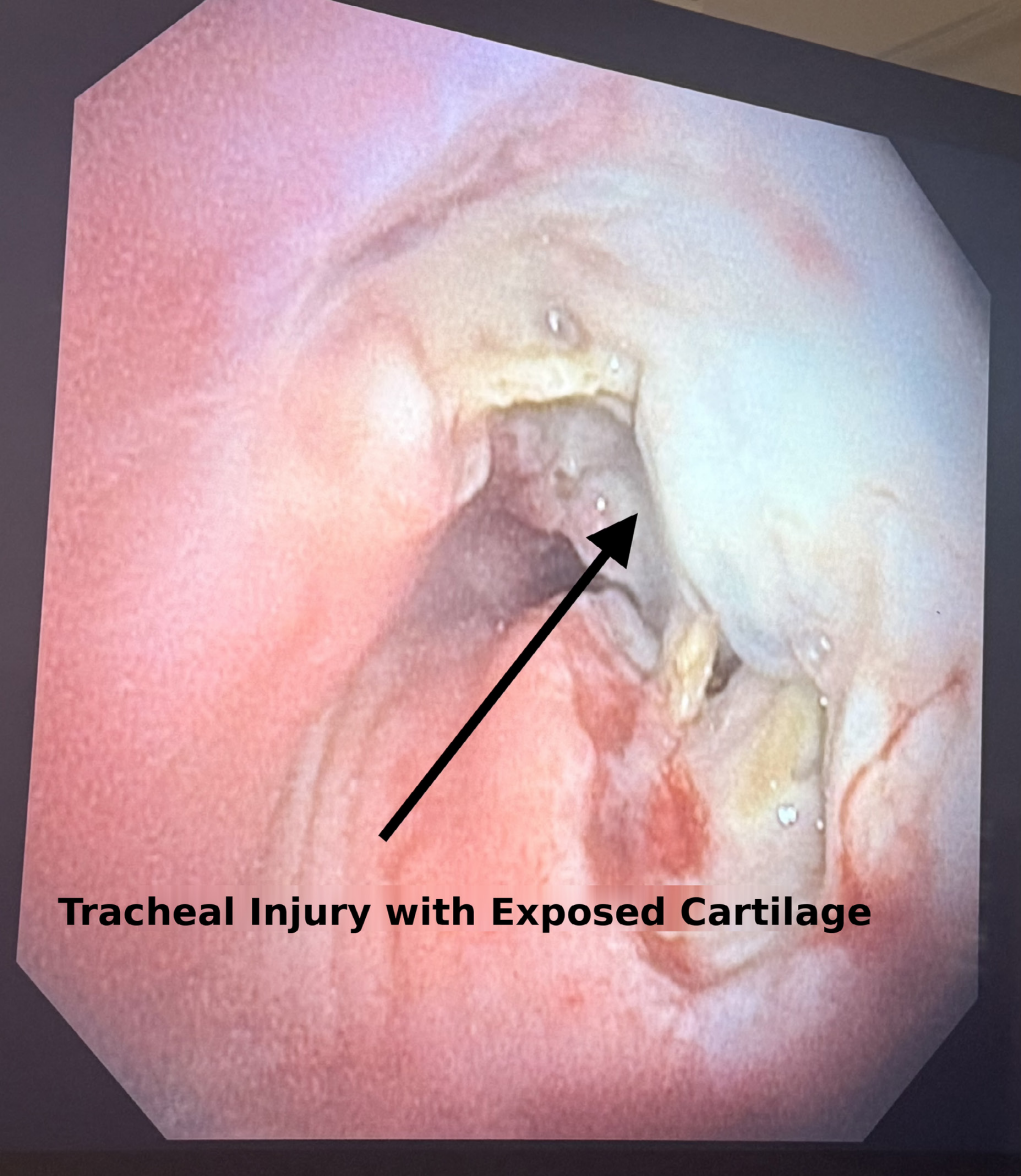
Flexible bronchoscopy image (March 4, 2026) demonstrating radiation‐induced tracheal chondroradionecrosis. Circumferential mucosal sloughing with exposed, devitalized tracheal cartilage rings is evident (arrow), involving the right anterior, right lateral, and left lateral tracheal walls. Areas of mucosal haemorrhage and ulceration at viable–necrotic junctions are visible. The posterior membranous wall was partially spared (not shown). Bronchoscopic findings were obtained approximately 19 months after completion of consolidative thoracic radiotherapy (6000 cGy in 15 fractions). The complete procedure, including intralesional triamcinolone acetonide injection technique, is demonstrated in Video 1.

**Video 1:** Complete procedure, including intralesional triamcinolone acetonide injection technique. Video content can be viewed at https://onlinelibrary.wiley.com/doi/10.1002/rcr2.70578.

## Author Contributions

**Venkatkiran Kanchustambham:** conception and design, acquisition of clinical data and bronchoscopic video, interpretation of findings, drafting and critical revision of the manuscript, final approval of the submitted version.

## Funding

The author has nothing to report.

## Consent

The author declares that written informed consent was obtained for the publication of this manuscript and accompanying images using the consent form provided by the Journal.

## Conflicts of Interest

The author declares no conflicts of interest. V.K. is an Editorial Board member of Respirology Case Reports and the author of this article. He was excluded from all editorial decision‐making related to the acceptance of this article for publication.

## Data Availability

The data that support the findings of this study are available on request from the corresponding author. The data are not publicly available due to privacy or ethical restrictions.

## References

[1] H. A. Gaissert , H. C. Grillo , M. B. Shadmehr , et al., “Laryngotracheal Reconstruction for Radiation‐Induced Tracheal Stenosis,” Journal of Thoracic and Cardiovascular Surgery 127, no. 1 (2004): 39–47.

[2] S. Delanian and J. L. Lefaix , “The Radiation‐Induced Fibroatrophic Process: Therapeutic Perspective via the Antioxidant Pathway,” Radiotherapy and Oncology 73, no. 2 (2004): 119–131. 15542158 10.1016/j.radonc.2004.08.021

